# P65 Targets FGFR1 to Regulate the Survival of Ovarian Granulosa Cells

**DOI:** 10.3390/cells8111334

**Published:** 2019-10-29

**Authors:** Xiaolong Yuan, Zhonghui Li, Yaru Kong, Yuyi Zhong, Yingting He, Ailing Zhang, Xiaofeng Zhou, Yao Jiang, Zhe Zhang, Hao Zhang, Jiaqi Li

**Affiliations:** 1 National Engineering Research Center for Swine Breeding Industry, Guangdong Provincial Key Lab of Agro-Animal Genomics and Molecular Breeding, College of Animal Science, South China Agricultural University, Guangzhou 510642, China; 2 Institute of Animal Biotechnology, Xinjiang Academy of Animal Science, Urumqi 830000, China; 3 College of Biology and Food Engineering/Development, Center of Applied Ecology and Ecological Engineering in Universities, Guangdong University of Education, Guangzhou 510303, China

**Keywords:** transcription factor p65, FGFR1, cell proliferation and apoptosis, ovarian granulosa cells

## Abstract

In female mammals, the abnormal apoptosis of ovarian granulosa cells (GCs) impairs follicular development and causes reproductive dysfunction. Many studies have indicated that the *FGFR1* gene of the PI3K signaling pathway and the p65 subunit of the transcription factor NF-κB may regulate the proliferation and apoptosis of GCs involved in follicular development. However, little is known about whether p65 regulates the transcription of *FGFR1*, as well as the biological effects of *p65* and *FGFR1* on the survival of GCs and follicular development. In porcine follicles and GCs, we found that *p65* and *FGFR1* were exclusively expressed in the GCs of follicles, and the mRNA and protein levels of *p65* and *FGFR1* significantly increased from small to large follicles. Both *p65* and *FGFR1* were found to activate the PI3K signaling pathway, and the expressions of proliferation markers (*PCNA* and *MKI67*) and the anti-apoptotic gene *BCL2* were significantly increased by *p65* and *FGFR1*. Furthermore, both *p65* and *FGFR1* were observed to promote cell proliferation and inhibit the cell apoptosis of GCs, and p65 was confirmed to bind at the −348/−338 region of *FGFR1* to positively regulate its transcription. Moreover, p65 was further found to enhance the pro-proliferation and anti-apoptotic effects of *FGFR1*. Taken together, p65 may target the −348/−338 region of *FGFR1*, promote the transcription of *FGFR1*, and enhance the pro-proliferation effect and anti-apoptotic effect of *FGFR1* to facilitate the growth of follicles. This study will provide useful information for further investigations on the p65-mediated-FGFR1 signaling pathway during folliculogenesis in mammals.

## 1. Introduction

In female mammals, the follicles, which consist of an oocyte surrounded by somatic cell components, i.e., granulosa cells (GCs) and theca cells [1,2] are the fundamental functional units of ovaries. The main functions of follicles are to produce mature oocytes for the propagation of the species and to synthesize the estrogens that support sexual responses [1,2]. During folliculogenesis, which includes selection, recruitment, dominance, and ovulation, the follicles dynamically progress through the primordial, primary, secondary, antral, and mature stages, with most follicles falling into atretic stages [1,2,3]. It has been widely recognized that the survival of GCs plays an important role in deciding the fate of follicles during folliculogenesis [4,5]. A great number of studies have shown that abnormal apoptosis of GCs impairs follicular dynamics and then causes reproductive dysfunction [4,6] and polycystic ovary syndrome [7,8] in humans. However, the underlying molecular mechanisms regarding the survival of GCs involved in follicular development have been largely unexplored.

Previous studies have demonstrated that the fibroblast growth factors (FGFs) are indispensable for cellular processes such as cell proliferation, cell survival, and steroidogenesis in GCs [9,10]. The *FGF receptor 1* (*FGFR1*) gene, one of key genes of the PI3K signaling pathway, is the critically important regulator of follicular development [11,12]. In humans, loss of function of *FGFR1* has been reported to induce sexual immaturity and reproductive incompetence [13,14]. In buffalo, the mRNA and protein levels of *FGFR1* increase along with the growth of follicles [9,15]. In chickens, knockdown of the expression of *FGFR1* significantly inhibits the proliferation of GCs [16] and the growth of follicles [12]. Additionally, the transcription factor p65, one of the core components of transcription factor NF-κB, has been reported to regulate the expressions of genes involved in the survival of GCs and folliculogenesis [17,18]. In humans, *p65* has been identified to highly associate with polycystic ovary syndrome caused by the dysfunction of GCs [19]. In mice, *p65* promotes cell cycle entry in GCs [20]. In porcine atretic follicles caused by the excessive apoptosis of GCs, the expressions of *p65* is dramatically lower than that in healthy follicles [21]. These observations suggest that *FGFR1* and *p65* have an essential role in regulating the proliferation and apoptosis of GCs associated with follicular development.

Previously, we found that the promoter of *FGFR1* harbored several putative binding sites of p65. Therefore, we hypothesized that p65 might control the transcription of *FGFR1* and then regulate the proliferation and apoptosis of GCs. In this study, using gilts as the biological model, the expression patterns of *p65* and *FGFR1* during follicular development were first characterized, and then the biological effects of *p65* and *FGFR1* on cell survival, PI3K, and the apoptosis signaling pathway were investigated. The molecular regulations between *p65* and *FGFR1* were further identified. This study was the first report to explore the molecular relationship between *p65* and *FGFR1* in GCs, and these works will provide new insight into the effects of *p65* and *FGFR1* during follicular development in mammals.

## 2. Materials and Methods

### 2.1. Ethics Statement

The animal experiments were conducted according to the Regulations for the Administration of Affairs Concerning Experimental Animals (Ministry of Science and Technology, Beijing, China) and were approved by the Animal Care and Use Committer of South China Agricultural University, Guangzhou, China (Approval number: 2018B116).

### 2.2. Animals and Sample Preparation

Ovaries were collected from a single local commercial pig slaughterhouse in Guangzhou and transferred to our laboratory in phosphate-buffered saline containing penicillin (100 IU/mL) and streptomycin (100 μg/mL) (Invitrogen, Shanghai, China) at a storage temperature of 37 °C.

### 2.3. Culture of Porcine GCs In Vitro

The porcine ovarian GGs were cultured according to our previous studies [22,23]. Briefly, 5–7 mm follicles were punctured for the collection of GCs using a 1 mL syringe, and the isolated GCs were washed twice with phosphate-buffered saline preheated to 37 °C. The cells were seeded into 75 cm^2^ flasks and cultured at 37 °C under 5% CO_2_ in DMEM (Hyclone, Logan, UT, USA) containing 10% fetal bovine serum (Hyclone, Logan, UT, USA), 100 IU/mL penicillin, and 100 μg/mL streptomycin. When cells reached 80% coverage of the flask, cells were seeded into 24 well plates for further experiments.

### 2.4. Real-Time Quantitative PCR Analysis

When cells covered 80% of one well, pcDNA3.1-FGFR1, pcDNA3.1-p65, pcDNA3.1-Basic, si-p65, si-FGFR1, and the negative siRNA control were transfected into the cells for 48 h. At least three wells per group were collected for extraction of total RNA. The total RNA was extracted using TRIzol reagent (TaKaRa, Tokyo, Japan) and then reverse-transcribed using a PrimeScript RT Master Mix Synthesis Kit (TaKaRa, Tokyo, Japan) for mRNAs. The relative expression levels of mRNAs were quantified using Maxima SYBR Green qRT-PCR Master Mix (2×) (Thermo Scientific, Waltham, CF, UAS) in a LightCycler Real-Time PCR system (96 system, Roche Diagnostics Ltd., Basel, Switzerland). The expression level of *GAPDH* mRNAs was used as endogenous controls, and the fold changes were calculated using the 2^−ΔΔct^ method. The primer sequences are listed in Table 1.

### 2.5. Construction of the FGFR1 5′ Deletion and Luciferase Assay

The genomic DNA of porcine ovary tissues was extracted and used as a template in this study. PCR was performed by using PrimerSTAR^®^ (TaKaRa, Dalian, China) high fidelity enzyme to obtain the *FGFR1* promoter to a length of 2445 bp. The CAAT box, TATA box, GC box, and potential binding sites of p65 were predicted using AliBaba (http://gene-regulation.com/pub/programs/alibaba2/index.html), PROMO (http://alggen.lsi.upc.es/cgi-bin/promo_v3/promo/promoinit.cgi?dirDB=TF_8.3), and TFBIND (http://tfbind.hgc.jp). The putative binding sites of *C/EBPβ* concurrently predicted by all of those four tools were used for further analysis. The primers are presented in Table 2, and the PCR products were purified by gelatinization and the addition of an “A” tail with products to combine with pMD-18T, which were transformed into competent DH5α cells and were inoculated overnight on ampicillin-containing lysogeny broth plates at 37 °C. Furthermore, monoclonal bacteria bearing platelets were added to ampicillin of lysogeny broth medium to incubate overnight at 37 °C in a shaker. The bacteria were collected by centrifugation, the plasmids were extracted, and the correct plasmid for sequencing was named T-*FGFR1*. T-*FGFR1* was then used as a template, and seven different upstream primers were designed to amplify deletion fragments. The locations of the *FGFR1* deletion fragments were: P1 (−2044/+401), P2 (−1652/+401), P3 (−1069/+401), P4 (−815/+401), P5 (−700/+401), P6 (−377/+401), and P7 (−84/+401). Each deletion fragment was identified by Sanger sequencing. The correct plasmids for each deletion fragment containing *KpnI and BglII* cleavage sites (Table 2) were obtained. Simultaneously, each deletion fragment was cloned into the eukaryotic expression vector pGL3-Basic, which was digested with *KpnI* and BglII restriction endonucleases. According to Promega’s dual luciferase reporter assay kit (Promega, Madison, WI, USA), the BioTek Synergy 2 multifunctional microplate reader (BioTek, Winooski, VT, USA) was utilized for fluorescence detection. The ratio of the expression of firefly luciferase to renilla luciferase was the target fragment activity. The putative binding sites of *p65* were GGAAATTCCC/TTCCCTGGCC.

### 2.6. Cell Transfection, Proliferation, and the Apoptosis Assay

The coding sequences of *FGFR1* (Gene ID: 100153248, Accession Number: XM_005671767.3) and *p65* (Gene ID: 100135665, NM_001114281.1) were cloned into pcDNA3.1 (+) (ThermoFisher, Guangzhou, China) with the restrictive enzymes for *FGFR1* (*BamH*I and *Xba*I) and *p65* (*Kpn*I and *Xba*I). The sequences of primers for these coding sequences are shown in Table 2. The si-p65, si-FGFR1, and their respective negative control were synthesized and purified by RiboBio Co.Ltd. (Guangzhou, China). pcDNA3.1-FGFR1, pcDNA3.1-p65, si-p65, or si-FGFR1 was transfected into GCs by using Lipofectamine^TM^ 3000 Transfection Reagent (ThermoFisher, city, country) according to a previous study [24]. Briefly, when cells reached 70% coverage of one well, the plasmids were transfected into cells in antibiotic-free medium. The transfected cells were incubated at 37 °C for 6 h, after which the medium was completely replaced and the cells were maintained in culture until further treatment.

Cell proliferation assays were performed using a Cell-Light Edu Apollo 567 In Vitro Kit (RiboBio Co., Ltd., Guangzhou, China). GCs were seeded into 48 well plates one day prior to transfection. When the cells reached 70% coverage of one well, pcDNA3.1-FGFR1, pcDNA3.1-p65, pcDNA3.1-basic, si-FGFR1, si-p65, or siRNA-NC were transfected into the cells at different final concentrations for 48 h. The specific steps were: diluting the Edu solution 1: 1000 with cell culture medium to prepare 50 μM Edu medium, adding 150 μL of 50 μM Edu medium to each well for 2 h, discarding the culture medium, adding 150 μL of cell fixing solution (80% acetone) to each well for 30 min at room temperature, washing twice with PBS, adding 150 μL of penetrant (0.5% TritonX-100 in PBS) to permeabilize the cells and rinsing once with PBS, adding 150 μL of 1× Apollo staining solution, and incubating for 30 min at room temperature in the dark. DNA was then stained with 100 ng/mL DAPI (150 μL/well) for 20 min. Photographs of cells were taken by using a fluorescent microscope (EclipseE600, Nikon, Tokyo, Japan). All cells were stained in blue, and the cells in red were specifically proliferating and replicating. The overlays of red and green indicate the proliferation rate of target cells.

Analysis and detection of cell apoptosis were based on our previous studies [22,23]. Cell apoptosis assays were performed using an annexin V-FITC Apoptosis Detection Kit (BioVision, Milpitas, CA, USA) according to the manufacturer’s instructions. Briefly, GCs (1–5 × 10^5^ cells/well) were cultured in triplicate in 6 well plates for one day prior to transfection. When the cells reached 70% coverage of one well, the plasmids were transfected into the cells at different concentrations for 48 h. The cells were then harvested, washed twice with ice-cold PBS, and resuspended in 500 μL of binding buffer. Next, 1.25 μL of annexin V-FITC was added in the dark for 15 min at room temperature, then 1000× *g* centrifugation for 5 min was done at room temperature to remove the supernatant. The cells were gently resuspended with 0.5 mL precooling 1× solution, and 10 μL of PI (propidium iodide; 50 μg/mL, BD, New York, NY, USA) was added. Last, the cells were analyzed using a flow cytometer (Becton Dickinson Co., San Jose, CA, USA) using the FITC signal detector (FL1) and phycoerythrin emission signal detector (FL2). All experiments were performed at least three times. Cells in the lower right quadrant of figure are annexin-positive/PI-negative early apoptotic cells. The cells in the upper right quadrant are annexin-positive/PI-positive late apoptotic cells. Cells undergoing early and late apoptosis were identified as apoptotic cells.

### 2.7. Western Blot Analysis

The cells were harvested and analyzed for their expression levels of total p65 and FGFR1 using the anti-p65 and anti-FGFR1 antibodies (Cell Signaling Technology, Danvers, MA, USA). Protein concentrations were determined using a BCA Protein Assay Kit (Vigorous Bio-technology Beijing Co., Ltd., Beijing, China), and equal amounts of protein were separated by SDS-PAGE and electroblotted onto polyvinylidene difluoride membranes. The membranes were blocked with 5% nonfat milk in PBS containing a percentage of Tween-20 for 1 h, and then incubated with a primary antibody against hamartin (1:1000; Biorbyt, San Francisco, CA, USA) overnight at 4 °C. An anti-GAPDH antibody (1:3000; Sigma, St. Louis, MO, USA) or β-actin (1:2000; Sigma, St. Louis, MO, USA) was used as an internal control. After incubation with secondary antibodies for 1 h at room temperature, antibody-bound protein bands were visualized using an ECL-PLUS Kit (Amersham Biosciences, Piscataway, NJ, USA). The gray scale values of the bands were calculated using ImageJ software, which was freely downloaded from NIH. The relative protein expression levels of p65 and FGFR1 were normalized to β-Actin or GAPDH values. At least three replicates were conducted for each group.

### 2.8. Chromatin Immunoprecipitation Assay

The GCs were cross-linked when the cell density reached 80% of the wells. The original culture medium in the flask was then discarded, and the cells were sequentially treated with formaldehyde, glycine, and PBS-Halt Cocktail, and were then centrifuged to collect the cell pellet. The chromatin immunoprecipitation (ChIP) assay (Thermofisher, Rockford, IL, USA) was carried out according to the previously described ChIP protocol. The ChIP primer for the −348/−338 region of *FGFR1* is presented in Table 1 and was used to detect the binding extent of p65 and polymerase II. After immunoprecipitation, the p65 binding site was identified by PCR amplification. Total fragmented DNA was used as the input. The DNA marker was 500 bp.

### 2.9. Immunohistochemistry Assay

The immunohistochemical localizations of p65 and FGFR1 were evaluated by using the primary antibodies anti-p65 and anti-FGFR1 (Cell Signaling Technology). The immunohistochemical analysis was conducted according to a previous study [25]. Briefly, the large (5–7 mm) and small (3–5 mm) follicles were fixed at room temperature in 4% paraformaldehyde for 24 h, paraffin-embedded, and then serially sectioned at 5 μm. After deparaffinization, antigen retrieval, and blocking in normal goat serum, the sections were incubated overnight at 4 °C in anti-p65 and anti-FGFR1 (Cell Signaling Technology, USA), and the sections were washed and incubated with biotin-labeled goat anti-rabbit secondary antibody for 30 min. The sections were then washed and incubated with avidin–biotin–peroxidase for 10 min and counterstained with hematoxylin. Positive immunoreactivity was represented by dark brown straining, and negative immunoreactivity was represented by blue staining. Images were captured by using confocal microscopy (DM4000B, Leica, Wetzlar, Germany).

### 2.10. Statistical Analysis

All data are shown as mean ± standard deviation (SD) of repeated experiments. All experiments were independently repeated at least three times. Student’s t-test (two-tailed) was used to analyze the significance of mean differences in the data using R software (version 3.5.3). * indicates *p* < 0.05; ** indicates *p* < 0.01.

## 3. Results

### 3.1. Expression Patterns of p65 and FGFR1 in Follicles and GCs

**Figure 1:**
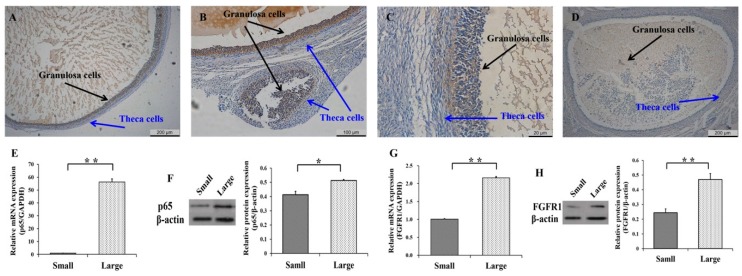
Expression patterns of *p65* and *FGFR1* in follicles and granulosa cells (GCs). The representations of immunohistochemical results of p65 (**A**,**B**) and FGFR1 (**C**,**D**) in large and small follicles, respectively. The relative mRNA (**E**) and protein (**F**) levels of *p65* in small and large follicles. The relative mRNA (**G**) and protein (**H**) levels of *FGFR1* in small and large follicles. * indicates *p* < 0.05; ** indicates *p* < 0.01. Scale bars: 200 μm for 5× figures; 100 μm for 10× figures; 20 μm for 20× figures.

To explore the biological functions of *p65* and *FGFR1* during the development of follicles, the expression patterns of *p65* and *FGFR1* in small (1–3 mm) and large (5–7 mm) follicles were first detected (Figure 1). In large (Figure 1A) and small (Figure 1B) follicles, the immunohistochemical results indicated that *p65* was likely to be exclusively expressed in GCs, as opposed to theca cells. Similar observations were made for *FGFR1* in the large (Figure 1C) and small (Figure 1D) follicles. Moreover, the mRNA (Figure 1E) and protein (Figure 1F) levels of *p65* were significantly higher in large follicles than in small follicles (*p* < 0.05), as were those of *FGFR1* (Figure 1G,H). These results indicated that *p65* and *FGFR1* might promote the development of follicles by affecting the function of GCs.

### 3.2. Pro-Proliferation and Anti-Apoptotic Effects of p65 on GCs

**Figure 2:**
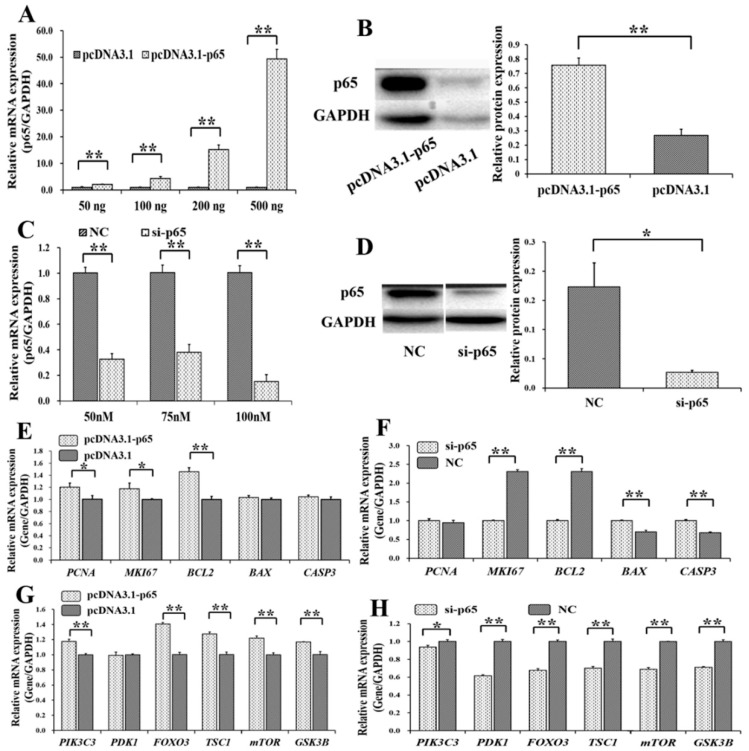
Biological effects of *p65* on apoptosis and PI3K signaling pathway. (**A**) The mRNA level of *p65* under different concentrations of pcDNA3.1-p65 plasmid; (**B**) the protein count of p65 was stimulated by 200 ng of pcDNA3.1-p65 plasmid; (**C**) the mRNA level of *p65* with different concentrations of siRNAs; (**D**) the protein count of *p65* was suppressed by 70 nM of si-p65. The relative mRNA expressions of genes from apoptosis signaling pathway after treatment by pcDNA3.1-p65 (**E**) and si-p65 (**F**); the relative mRNA expressions of genes from PI3K signaling pathway after treatment by pcDNA3.1-p65 (**G**) and si-p65 (**H**). * indicates *p* < 0.05; ** indicates *p* < 0.01. Data are represented as means ± SD. NC: negative control. Si-p65: specific siRNA for *p65*. PcDNA3.1-p65: pcDNA3.1 overexpression plasmid of *p65*.

To determine the biological effects of *p65* on the PI3K and apoptosis signaling pathway, overexpression plasmids and small interfering RNAs (siRNAs) of *p65* were built. We found that the mRNA level of *p65* increased with the concentration of pcDNA3.1-p65 (Figure 2A), and 200 ng of pcDNA3.1-p65 plasmid was selected for further use. Compared to the control group (pcDNA3.1), the protein level of p65 was significantly increased by 200 ng of pcDNA3.1-p65 (Figure 2B). The specific siRNA for p65 (si-p65) significantly inhibited the mRNA level of *p65* at 50, 75, and 100 nM (Figure 2C), compared to the negative control (NC). We selected 100 nM of si-p65 for further study on the considerations for cellular tolerance, and the protein count of p65 was significantly inhibited at 100 nM compared to the NC group (Figure 2D).

Among the genes from the apoptosis signaling pathway, pcDNA3.1-p65 was observed to significantly increase the mRNA levels of *PCNA* (*p* < 0.05), *MKI67* (*p* < 0.05), and *BCL2* (*p* < 0.01) (Figure 2E), and si-p65 significantly suppressed the mRNA levels of *MKI67* (*p* < 0.01) and *BCL2* (*p* < 0.01) but upregulated the mRNA levels of *BAX* (*p* < 0.01) and *CASP3* (*p* < 0.01) (Figure 2F). Among the genes of the PI3K signaling pathway, pcDNA3.1-p65 significantly increased the mRNA levels of *PIK3C3* (*p* < 0.01), *FOXO3* (*p* < 0.01), *TSC1* (*p* < 0.01), *mTOR* (*p* < 0.01), and *GSK3B* (*p* < 0.01) (Figure 2G). However, si-p65 significantly decreased the mRNA levels of *PIK3C3* (*p* < 0.05), *PDK1* (*p* < 0.01), *FOXO3* (*p* < 0.01), *TSC1* (*p* < 0.01), *mTOR* (*p* < 0.01), and *GSK3B* (*p* < 0.01) (Figure 2H).

**Figure 3:**
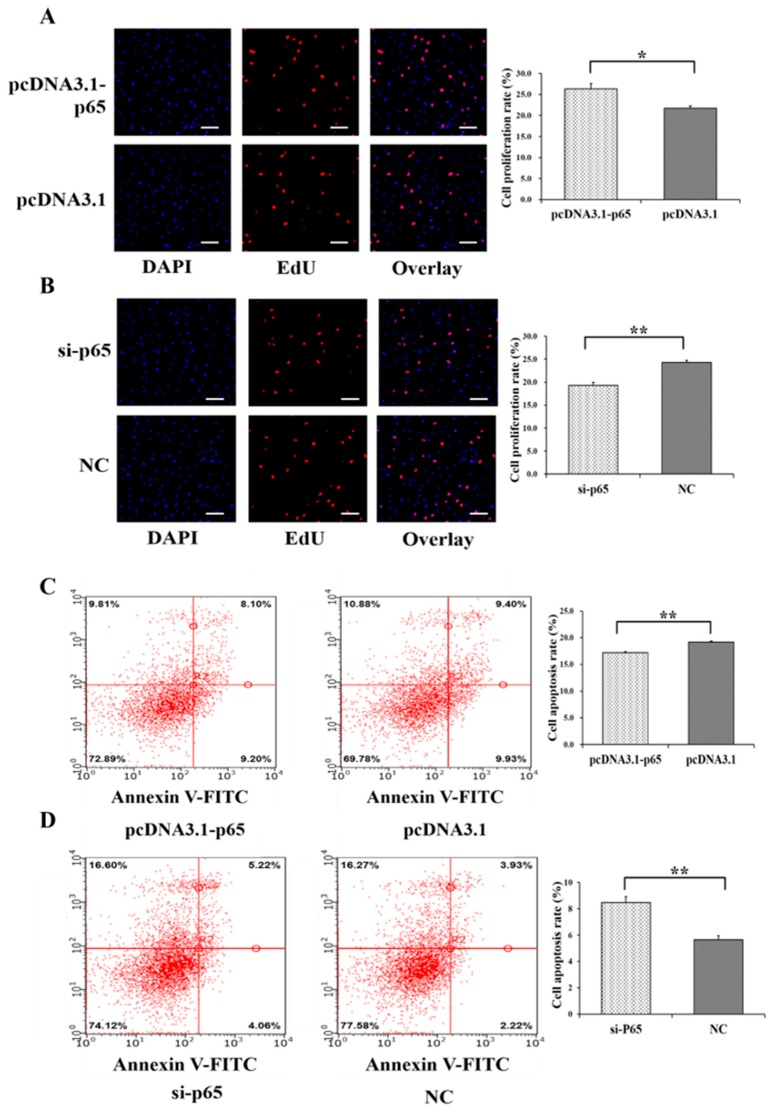
Biological effects of *p65* on cell proliferation and apoptosis of GCs. The cell proliferation rate of GCs after treatment by pcDNA3.1-p65 (**A**) and si-p65 (**B**). The cell apoptosis rate of GCs after treatment by pcDNA3.1-p65 (**C**) and si-p65 (**D**). * indicates *p* < 0.05; ** indicates *p* < 0.01. Data are represented as means ± SD. The scale bar represents 100 μm. NC: negative control. PcDNA3.1-p65: pcDNA3.1 overexpression plasmid of *p65*; si-p65: specific siRNA for *p65*.

Moreover, pcDNA3.1-65 was found to significantly increase the cell proliferation rate of GCs (*p* < 0.05) (Figure 3A), and si-65 significantly decreased the cell proliferation rate of GCs (*p* < 0.01) (Figure 3B). In addition, pcDNA3.1-p65 was observed to significantly depress the cell apoptosis rate of GCs (*p* < 0.01) (Figure 3C), and si-p65 significantly promoted cell apoptosis in GCs (*p* < 0.01) (Figure 3D).

### 3.3. p65 Targets FGFR1 and Promotes the Expression of FGFR1

**Figure 4:**
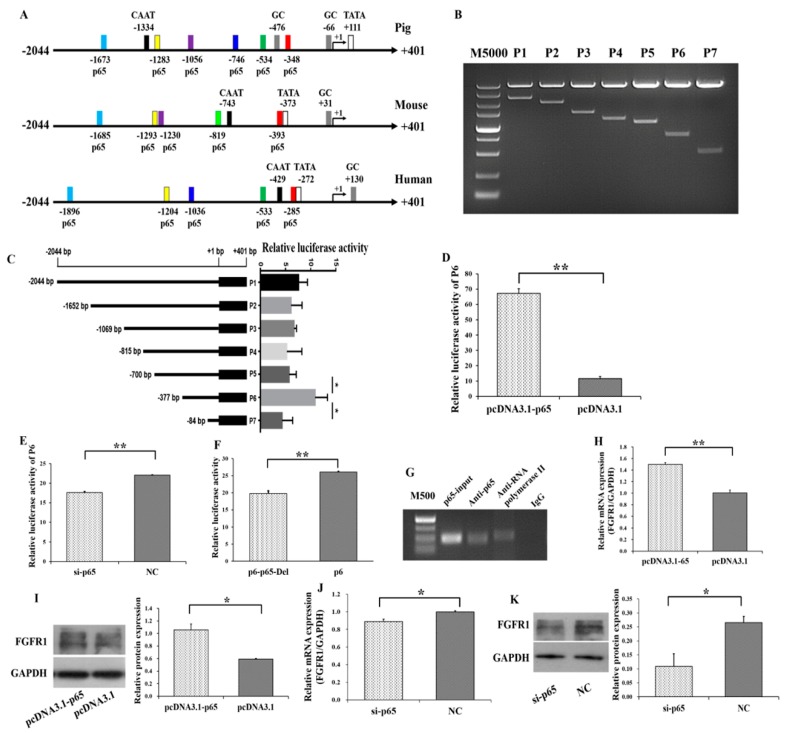
p65 binds to the −348/−338 region of *FGFR1* and positively regulate the expression of *FGFR1*. (**A**) The putative binding sites of p65 on the promoter of *FGFR1*; (**B**) PCR products of 5′ deletion fragments from promoter of *FGFR1*; (**C**) the relative luciferase activity of *FGFR1* promoter after 5′ deletion of the putative binding sites of p65; (**D**) the relative luciferase activity of P6 with overexpression of p65; (**E**) the relative luciferase activity of P6 with interfering with expression of p65; (**F**) the relative luciferase activity of P6 after specific deletion of −348/−338; (**G**) confirmation of p65 binding at −348/−338 by chromatin immunoprecipitation (ChIP) assay. The relative mRNA (**H**) and protein (**I**) levels of FGFR1 stimulated by pcDNA3.1-p65; the relative mRNA (**J**) and protein (**K**) levels of FGFR1 inhibited by si-p65. * indicates *p* < 0.05; ** indicates *p* < 0.01. Data are represented as means ± SD. CAAT: CAAT box; TATA: TATA box: GC: GC box; M5000: DNA markers of 5000 bp. M500: DNA markers of 500 bp. Si-p65: specific siRNA for *p65*; NC: negative control. pcDNA3.1-p65: pcDNA3.1 overexpression plasmid of *p65*.

Several putative binding sites of p65 on the promoter of *FGFR1* in pigs, mice and humans were predicted (Figure 4A). The potential binding sites of p65 were −1673/−1663, −1283/−1273, −1056/−1046, −746/−736, −534/−524, and −348/−338 on the promoter of *FGFR1* in pigs (Figure 4A). To further explore the molecular mechanism for p65 regulating the expression of *FGFR1*, 5′ deletion and gene reporter assay were constructed for *FGFR1* in pigs. The 5′ deletion fragments of P1 (−2044/+401), P2 (−1652/+401), P3 (−1069/+401), P4 (−815/+401), P5 (−700/+401), P6 (−377/+401), and P7 (−84/+401) were amplified and cloned into the eukaryotic expression vector pGL3-Basic (Figure 4B). Compared to P1, the relative luciferase activities of P2, P3, P4, and P5 did not show significant changes (Figure 4C) with deletion of −1673/−1663, −1283/−1273, −1056/−1046, and −746/−736, respectively. After deletion of −534/−524 (P5), the relative luciferase activity of P6 was significantly increased. However, after deletion of −348/−338 (P6), the relative luciferase activity was significantly decreased (P7). The −348/−338 region of *FGFR1* is surrounded by the GC and TATA box. Moreover, the expression patterns of p65 and *FGFR1* were positively correlated in small and large follicles (Figure 1E–H), indicating that P6 might harbor the exact binding site of p65.

Furthermore, the relative luciferase activity of P6 was significantly increased by pcDNA3.1-p65 (Figure 4D) and was significantly decreased by si-65 (Figure 4E). To further validate *FGFR1* as a target of p65, the −348/−338 region of P6 was further specifically deleted, and this deletion significantly decreased the relative luciferase activity (Figure 4F). The results of the chromatin immunoprecipitation (ChIP) assay on P6 further confirmed that p65 binds at the −348/−338 region of *FGFR1* (Figure 4G). To identify whether p65 regulates the expression of *FGFR1*, the expression of *FGFR1* was determined after transfection with pcDNA3.1-p65 or si-p65 into GCs. The mRNA (*p* < 0.01, Figure 4H) and protein (*p* < 0.05, Figure 4I) levels of *FGFR1* were significantly increased by pcDNA3.1-65, and the mRNA (*p* < 0.05, Figure 4J) and protein (*p* < 0.05, Figure 4K) levels of *FGFR1* could be significantly decreased by si-65. These results indicated that p65 might upregulate the transcription and expression of *FGFR1* in GCs.

### 3.4. Pro-Proliferation and Anti-Apoptotic Effects of FGFR1 on GCs

**Figure 5:**
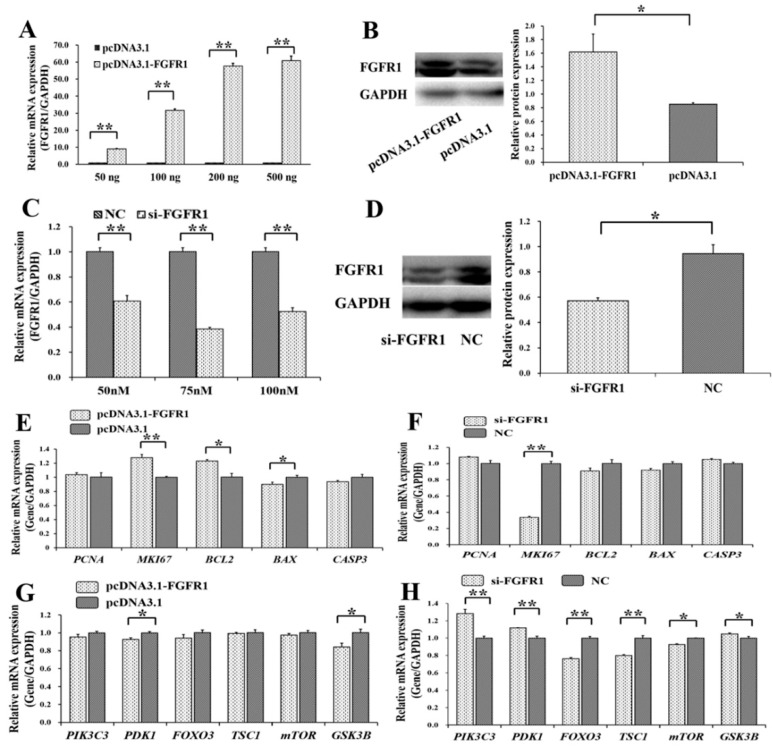
Biological effects of *FGFR1* on apoptosis and PI3K signaling pathway. (**A**) The mRNA level of *FGFR1* under different concentrations of pcDNA3.1-FGFR1 plasmid; (**B**) the FGFR1 protein count was stimulated by 200 ng of pcDNA3.1-FGFR1 plasmid; (**C**) the mRNA level of *FGFR1* with different concentrations of siRNAs; (**D**) the FGFR1 protein count was suppressed by 70 nM of si-FGFR1. The relative mRNA expressions of genes from apoptosis signaling pathway after treatment by pcDNA3.1-FGFR1 (**E**) and si-FGFR1 (**F**); The relative mRNA expressions of genes from PI3K signaling pathway after treatment by pcDNA3.1-FGFR1 (**G**) and si-FGFR1 (**H**). * indicates *p* < 0.05; ** indicates *p* < 0.01. Data are represented as means ± SD. NC: negative control. Si-FGFR1: specific siRNA for *FGFR1*. PcDNA3.1-FGFR1: pcDNA3.1 overexpression plasmid of *FGFR1*.

To determine the biological effects of *FGFR1* on the PI3K and apoptosis signaling pathways, as well as cell survival, overexpression plasmids and specific siRNAs of *FGFR1* were built and created (Figure 5). In GCs, the mRNA level of *FGFR1* was increased along with the concentration of pcDNA3.1-FGFR1 (Figure 5A). We selected 200 ng of pcDNA3.1-FGFR1 plasmid for further use, and 200 ng of pcDNA3.1-FGFR1 was observed to significantly increase the protein level of FGFR1, compared to pcDNA3.1-basic (Figure 5B). Quantities of 50 nM, 75 nM, and 100 nM of siRNA specific for FGFR1 (si-FGFR1) were found to significantly decrease the mRNA level of *FGFR1* (Figure 5C), and 75 nM of si-FGFR1 was selected for further use based on considerations of cellular tolerance. We found that 75 nM of si-FGFR1 significantly decreased the level of FGFR1 protein, compared to the NC group (Figure 5D).

**Figure 6:**
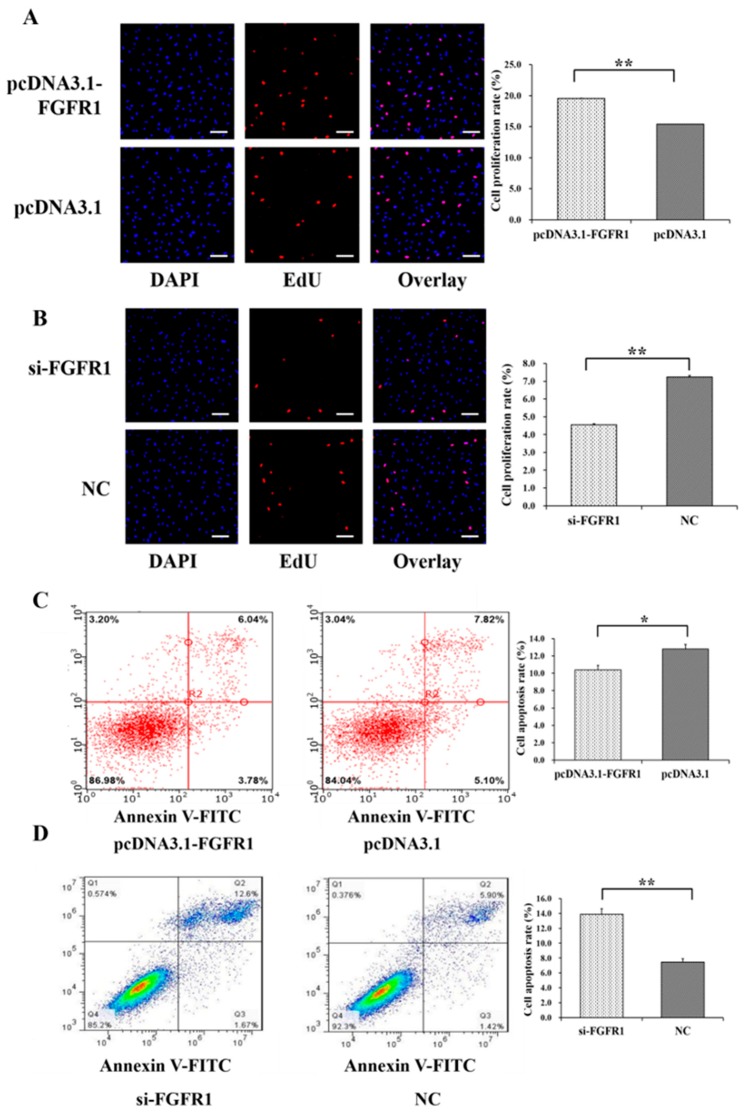
Biological effects of *FGFR1* on cell proliferation and apoptosis of GCs. The cell proliferation rate of GCs after treatment by pcDNA3.1-FGFR1 (**A**) and si-FGFR1 (**B**). The cell apoptosis rate of GCs after treatment by pcDNA3.1-FGFR1 (**C**) and si-FGFR1 (**D**). * indicates *p* < 0.05; ** indicates *p* < 0.01. Data are represented as means ± SD. The scale bar represents 100 μm. NC: negative control; PcDNA3.1-FGFR1: pcDNA3.1 overexpression plasmid of *FGFR1*; Si-FGFR1: specific siRNA for *FGFR1*.

Among the genes of the apoptosis signaling pathway, pcDNA3.1-FGFR1 was found to significantly increase the mRNA levels of *MKI67* (*p* < 0.05) and *BCL2* (*p* < 0.01) but significantly decrease the mRNA levels of *BAX* (*p* < 0.05) (Figure 5E), and si-p65 significantly decreased the mRNA levels of *MKI67* (*p* < 0.01) (Figure 5F). Among the genes of the PI3K signaling pathway, pcDNA3.1-FGFR1 was observed to significantly decrease the mRNA levels of *PDK1* (*p* < 0.05) and *GSK3B* (*p* < 0.05) (Figure 5G), and si-FGFR1 was observed to significantly increase the mRNA levels of *PIK3C3* (*p* < 0.01), *PDK1* (*p* < 0.01), and *GSK3B* (*p* < 0.05) but significantly decrease the mRNA levels of *FOXO3* (*p* < 0.01), *TSC1* (*p* < 0.01), and *mTOR* (*p* < 0.05) (Figure 5H). Moreover, pcDNA3.1-FGFR1 significantly increased the cell proliferation rate (Figure 6A), and si-FGFR1 significantly decreased the cell proliferation rate in GCs (Figure 6B). pcDNA3.1-FGFR1 significantly depressed the cell apoptosis rate (Figure 6C), and si-FGFR1 significantly increased the cell apoptosis rate (Figure 6D) in GCs.

### 3.5. p65 Enhances the Pro-Proliferation and Anti-Apoptotic Effects of FGFR1 in GCs

**Figure 7:**
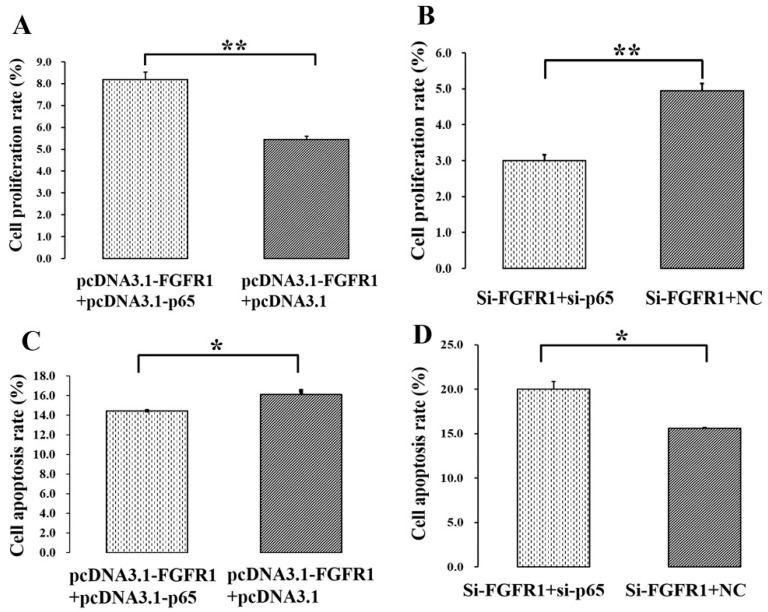
*p65* enhances the pro-proliferation and anti-apoptotic effects of *FGFR1* in GCs. (**A**) PcDNA3.1-p65 increased the cell proliferation rate of pcDNA3.1-FGFR1 in GCs; (**B**) si-p65 decreased the cell proliferation rate of si-FGFR1 in GCs; (**C**) pcDNA3.1-p65 depressed the cell apoptosis rate of pcDNA3.1-FGFR1 in GCs; (**D**) si-p65 stimulated the cell apoptosis rate of si-FGFR1 in GCs. Data are represented as means ± SD. NC: negative control; PcDNA3.1-FGFR1: pcDNA3.1 overexpression plasmid of *FGFR1*; PcDNA3.1-p65: pcDNA3.1 overexpression plasmid of *p65*; si-FGFR1: specific siRNA for *FGFR1*; si-p65: specific siRNA for *p65*; NC: negative control; * indicates *p* < 0.05; ** indicates *p* < 0.01.

To further investigate the biological effects of *p65* on the functions of *FGFR1*, pcDNA3.1-FGFR1, pcDNA3.1-p65, si-FGFR1 and si-p65 were co-transfected into GCs. For cell proliferation, pcDNA3.1-FGFR1 + pcDNA3.1-p65 was found to be markedly higher than pcDNA3.1-FGFR1 + pcDNA3.1 (Figure 7A), and si-FGFR1 + si-p65 was significantly lower than si-FGFR1 + NC (Figure 7B). These results indicated that *p65* might enhance the pro-proliferation effect of *FGFR1* in GCs. For cell apoptosis, pcDNA3.1-FGFR1 + pcDNA3.1-p65 was found to be significantly lower than pcDNA3.1-FGFR1 + pcDNA3.1 (Figure 7C), and si-FGFR1 + si-p65 was significantly higher than si-FGFR1 + NC (Figure 7D). These results implied that *p65* might enhance the anti-apoptotic effect of *FGFR1* in GCs.

## 4. Discussion

In mammals, previous studies have suggested that the survival of GCs decides the fate of follicles [4,5]. It is well known that the positive proliferation of GCs facilitates the development of follicles, but that the excessive apoptosis of GCs impairs the growth of follicles, induces the atresia of follicles [4,5,26], and even results in delayed sexual maturation [27]. The transcription factor p65, one core subunit of NF-κB, regulates the proliferation and apoptosis of many ovarian cells [18,28]. The *FGFR1* gene is the critically important regulator of the proliferation of GCs [9] and follicular development [11,12]. In this study, we found that p65 targeted at the −348/−338 region of *FGFR1*, promoted the transcription of *FGFR1*, and enhanced the pro-proliferation and anti-apoptotic effect of *FGFR1* to facilitate the growth of follicles. This study will provide useful information for further investigations on the p65-mediated-*FGFR1* signal pathway during folliculogenesis in mammals. Generally, once p65 has formed heterodimers with one of the other NF-κB proteins, e.g., p50, the NF-κB signaling pathway is activated. Then released NF-κB will transmigrate from translocation into the nucleus and bind to DNA to regulate the expression of target genes [29]. However, many studies have suggested that p65 is different from p50 in regulating the transcription of targeted genes [18,30]. For example, it is the p65/p65 or p50/p50 homodimer, not the p65/p50 heterodimer, that directly regulates the expression of target genes to mediate cell survival in humans [30,31], and the homodimer of p50/50 or p65/p65 controls basic ovarian functions in pigs [17]. These observations support the idea that the p65/p65 homodimer may control the expression of *FGFR1* to regulate the survival of GCs in pigs.

In this study, the mRNA and protein levels of *FGFR1* were significantly increased by p65 (Figure 4H–K), and the expression pattern of *FGFR1* was in line with that of p65 in small and large follicles (Figure 1E–H). These results indicated that p65 might upregulate the expression of *FGFR1* in porcine GCs. To further investigate whether p65 regulates the transcription of *FGFR1*, several 5′ deletion fragments of the promoter of *FGFR1* and the luciferase report assay were created (Figure 4B,C). After deletion of the −348/−338 region of P6, the relative luciferase activity of P7 was significantly decreased (Figure 4C), and p65 significantly increased the relative luciferase activity of P6 (Figure 4D,E). After specific deletion of the −348/−338 region of P6, the relative luciferase activity of P6 significantly decreased (Figure 4F). Moreover, the results of the ChIP assay on P6 confirmed that p65 binds at the −348/−338 region of *FGFR1* (Figure 4G), and *p65* was observed to enhance the pro-proliferation effect and anti-apoptotic effect of *FGFR1* (Figure 7). These results demonstrated that p65 targeted the −348/−338 region of *FGFR1* to positively regulate the expression of *FGFR1* in porcine GCs. Additionally, there were also several potential binding sties on the promoter of *FGFR1* in humans, pigs, and mice (Figure 4A), and the locations of these potential binding sties were likely to converge among humans, mice, and pigs (Figure 4A). Moreover, the confirmed binding site of p65 (−348/−338 region) on the promoter of *FGFR1* in pigs was the nearest one to the transcription start site and was surrounded by the TATA box and GC box (Figure 4A). The nearest putative binding site of p65 to the transcription start site being surrounded by the TATA box and GC box was also observed in humans or mice (Figure 4A). These observations indicated that p65 might regulate the expression of *FGFR1* in mammals.

To explore the biological functions of *p65* and *FGFR1* on cell survival of GCs, the expressions of genes from the apoptosis signaling pathway were detected after knockdown or overexpression of *p65* (Figure 2) and *FGFR1* (Figure 5) in GCs. Among the apoptosis-related genes, the mRNA levels of the proliferation markers *PCNA* and *MKI67* and the anti-apoptotic gene *BCL2* were significantly increased by overexpression of *p65* (Figure 2 E), and the mRNA levels of *MKI67* and *BCL2* were significantly decreased by interfering with the expression of *p65* (Figure 2F). Furthermore, the mRNA levels of *MKI67* and *BCL2* were also significantly upregulated by overexpression of *FGFR1* (Figure 5E), and the mRNA level of *MKI67* was significantly downregulated by interfering with the expression of *FGFR1* (Figure 5F). Previous studies have suggested that *MK167* presents exclusively in proliferating cells and servers as a marker for proliferation [32]. In mouse GCs, the expression patterns of *MKI67* were coupled with the patterns of *follicle-stimulating hormone receptor* [33]. Compared to wild-type mice, the expression of *MKI67* in GCs is significantly lower than in GC-specific steroidogenic factor 1 deficiency mice with impaired follicle development and infertility [34]. *BCL2*, known to be an apoptosis regulator, has been reported to block the apoptotic death [35] of GCs in bovines [36] and humans [37].

Additionally, the mRNA levels of apoptotic activators such as *BAX* and *CASP3* were significantly upregulated by interfering with the expression of p65 (Figure 2F), and the mRNA levels of *BAX* and *CASP3* were upregulated by interfering with expression of *FGFR1* (Figure 5F), although the change in the expression of *CASP3* was not statistically significant (Figure 5F). It is well known that *BAX* functions as an apoptotic activator and apoptosis marker. The induced expression of *BAX* is likely to trigger cell apoptosis in GCs of mice [38,39]. Higher expressions of *CASP3*, one of the pro-apoptotic genes, induce the apoptosis of GCs in bovines [36]. Downregulation of the pro-apoptotic *CASP3* gene inhibits the apoptosis of GCs in pigs [40]. The mRNA level of *CASP3* in GCs is negatively associated with the number of preovulatory follicles in humans, indicating that the accumulation of *CASP3* impairs the development of follicles [41]. Furthermore, the cell proliferation rate was significantly promoted by p65 (Figure 3A,B) or FGFR1 (Figure 6A,B), and the cell apoptosis rate was significantly suppressed by p65 (Figure 3C,D) or FGFR1 (Figure 6C,D). These observations suggested that both *p65* and *FGFR1* might promote cell proliferation and inhibit cell apoptosis in porcine GCs.

Previous studies have indicated that the PI3K signaling pathway plays an important role in regulation of cell survival of GCs [42,43]. Because of the *FGFR1* gene from the PI3K signaling pathway, the mRNA levels of some other genes from the PI3K signaling pathway were also detected after knockdown or overexpression of *p65* (Figure 2G,H) and *FGFR1* (Figure 5G,H) in GCs. We found that the mRNA levels of *PDK1* and *GSK2B* were significantly downregulated by overexpression of *FGFR1* (Figure 5G), and the mRNA levels of *PIK3C3*, *PDK1*, *GSK3B* were significantly upregulated but that of *FOXO3*, *TSC1* and *mTOR* were significantly downregulated by interference with the expression of *FGFR1* (Figure 5H). Furthermore, the mRNA levels of *PIK3C3*, *FOXO3*, *TSC1*, *mTOR*, and *GSK3B* were significantly increased by overexpression of *p65* (Figure 2G) but were significantly decreased by interfering with the expression of *p65* (Figure 2H). The mRNA level of *PDK1* was also significantly decreased by interfering with the expression of *p65* (Figure 2H). A previous study has suggested that a high expression level of *PIK3C* is associated with follicular development and subfertility in mice [44]. A high mRNA level of *PDK1* depresses cell apoptosis and affects cell entry in porcine GCs [45]. *FOXO3*-specific-depletion in GCs decreases both cell proliferation and apoptosis of GCs and disrupts follicular growth in mice [46], and a lower mRNA expression of *FOXO3* in GCs is involved in infertility in women [47]. *TSC1*-specific-knockout in GCs stimulates the development of follicles in mice [48], and moreover, interfering with the expression of *TSC1* significantly promotes cell proliferation and inhibits cell apoptosis in porcine GCs [23]. The *mTOR* gene has been reported to mediate cellular responses to stress such as DNA damage and to determine the fate of GCs in mice [49]. Inhibition of the expression of *GSK3B* represses the growth of GCs in humans [50]. These results suggest that both *p65* and *FGFR1* might regulate the biological functions of GCs during follicular development via the PI3K signaling pathway.

## Figures and Tables

**Table 1:** Primers of real-time PCR (RT-PCR), chromatin immunoprecipitation (ChIP) assay, and coding sequence cloning.

Name	Sequence	Product (bp)	Accession Number
CDS-FGFR1	F: CGGGATCCGCTCACCAACGAGGATCAA R: GCTCTAGAGTCACAGCTAAGGATGGAGT	2597	XM_005671767.3
CDS-P65	F: GGGGTACCATGGACGACCTCTTCCCCCT R: GCTCTAGATTAGGAGCTGATCTGACTCA	1662	NM_001114281.1
ChIP-FGFR1	F: GACTCAGTTTAGCGCATTGC R: GAGAAAAGTCCTCAGGCTCC	92	XM_005671767.3
ChIP-GAPDH	F: GATGTCCTGAGCCCCTACAG R: GGTAGGTGATGGGGACTGAG	102	NM_001206359.1
qRT-PCR-FGFR1	F: GGCTACAAGGTCCGTTATG R: CAATCTTACTCCCATTCACC	289	XM_005671767.3
qRT-PCR-P65	F: CATGCGCTTCCGCTACAAG R: GGTCCCGCTTCTTTACACAC	284	NM_001114281.1
qRT-PCR-GAPDH	F: TCGGAGTGAACGGATTTG R: TCACCCCATTTGATGTTGG	250	NM_001206359.1
qRT-PCR-PCNA	F: ATGCAGACACCTTGGCACTA R: TTTCCGAGTTCTCCACTTGC	247	NM_001291925.1
qRT-PCR-CASP3	F: AGGAGCAGTTTTATTTGCGTGC R: AGTCCCACTGTCCGTCTCAA	204	NM_214131.1
qRT-PCR-GSK3B	F: TAGTCGAGCCAAACAGACGC R: ATTGGGTTCTCCTCGGACCA	202	NM_001128443.1
qRT-PCR-BAX	F: AGCGCATTGGAGATGAACTG R: AAGTAGAAAAGCGCGACCAC	157	XM_003127290.5
qRT-PCR-BCL2	F: TTGCCGAGATGTCCAGCCAG R: TCAGTCATCCACAGGGCGAT	202	XM_021099593.1
qRT-PCR-PIK3C3	F: AACGGGCTTTGAGATAGTGAA R: AAGTTGCTTGGTTGGTGGATA	184	NM_001012956.2
qRT-PCR-PDK1	F: ACATGTACTCCACTGCACCC R: AGGCGTGATATGGGCAATCC	91	NM_001159608.1
qRT-PCR-FOXO3	F: ACAAACGGCTCACTCTGTCCCA R: GAACTGTTGCTGTCGCCCTTATC	85	NM_001135959.1
qRT-PCR-TSC1	F: AGCCGTCGTGGAAGCATAAG R: GTGCAGGTGGACGAGATAGA	245	XM_021070814.1
qRT-PCR-mTOR	F: CCGTCATCCCCTTATCCACC R: GGTCGTAGTCTGGAGCCATC	153	XM_003127584.6
qRT-PCR-MKI67	F: ACAACAGGAGGAGGAAGTGCT R: TTGACCTAGACGCGGGGAT	300	XM_021073740.1

**Table 2:** Primers for 5′ deletion fragments of *FGFR1* promoter.

Name	Sequences	Product (bp)	Accession Number
p1 (−2044/+401)	F: GG***GGTACC***AAATTAGGGGACAAGGTTATCT	2445	XM_005671767.3
	R: GA***AGATCT***GAGTTGGCGGAAAAGTTGGG		
p2 (−1652/+401)	F: GG***GGTACC***ATAGCCTGATTCCTCAAGTCTG	2053	XM_005671767.3
	ditto		
p3 (−1069/+401)	F: GG***GGTACC***GTTGCGCTGCCTGTGGTGTA	1470	XM_005671767.3
	ditto		
p4 (−815/+401)	F: GG***GGTACC***ACTTCAGGGCTACAGCGTCT	1216	XM_005671767.3
	ditto		
p5 (−700/+401)	F: GG***GGTACC***AGCCAGAACGCAGGAAAGGA	1101	XM_005671767.3
	ditto		
p6 (−377/+401)	F: GG***GGTACC***GACTCAGTTTAGCGCATTGC	778	XM_005671767.3
	ditto		
p7 (−84/+401)	F: GG***GGTACC***GCTTCGGCTCCATTGTTCC	485	XM_005671767.3
	ditto		
P6 deletion	F: GGCGACCTCG****CCGCGGGCGCGCGCTGCATC	748	XM_005671767.3
	R: GA***AGATCT***GAGTTGGCGGAAAAGTTGGG		
P6 deletion	F: GG***GGTACC***GACTCAGTTTAGCGCATTGC	50	
	R: GATGCAGCGCGCGCCCGCGG****CGAGGTCGCC		

* Underlined indicates base protection. Bold and italics indicate enzyme-cutting sites.

## Data Availability

The datasets used in the current study are available from the corresponding author on reasonable request.
